# Association between frailty status and risk of chronic lung disease: an analysis based on two national prospective cohorts

**DOI:** 10.1007/s40520-024-02867-8

**Published:** 2024-11-09

**Authors:** Gui-Yu Feng, Jing-Xiao Li, Guo-Sheng Li, Jun Liu, Xiang Gao, Guan-Qiang Yan, Nuo Yang, Tao Huang, Hua-Fu Zhou

**Affiliations:** 1 Department of Cardiothoracic Surgery, The First Affiliated Hospital of Guangxi Medical University, Guangxi Zhuang Autonomous Region, No. 6, Shuangyong Road, Nanning, 530021 P. R. China; 2 Department of Cardiothoracic Vascular Surgery, The Affiliated Hospital of Youjiang Medical University for Nationalities, Guangxi Zhuang Autonomous Region, Baise, 533000 P. R. China

**Keywords:** Frailty, Chronic lung disease, Prospective, Cohort

## Abstract

**Background:**

The association between the frailty index (FI) and the risk of chronic lung diseases (CLDs) remains unexplored, warranting further research.

**Methods and materials:**

This study investigated the relationship between FI and CLD risk using data from the China Health and Retirement Longitudinal Study (CHARLS) and English Longitudinal Study of Ageing (ELSA), comprising a combined sample of 9642 individuals. Propensity score weighting was used to ensure similar distribution of covariates across FI groups. The Wilcoxon rank-sum test was used to analyze differences in FI scores between groups with and without CLD. Kaplan–Meier curves and Cox regression analysis were employed to explore the association between frailty status and CLD incidence, with sensitivity analyses conducted for validation.

**Results:**

Higher FI scores were significantly associated with increased CLD risk in both cohorts (*p* < .05). Kaplan–Meier survival and Cox regression analyses indicated that frail individuals have a significantly elevated risk of CLD compared to robust individuals, particularly in certain subgroups (e.g., female) within the CHARLS cohort (*p* < .05). The ELSA cohort yielded similar results (*p* < .05), affirming FI as a strong predictor of CLD. Additional risk factors identified included age, smoking, and unmarried status (*p* < .05). Frail individuals consistently exhibited the highest risk in both cohorts (CHARLS HR = 1.54, *p* = .003; ELSA HR = 6.64, *p* < .001). The sensitivity analysis did not substantially alter the significant associations.

**Conclusion:**

These findings emphasize the critical role of frailty in the development of CLD, suggesting that targeted interventions could reduce CLD risk.

**Supplementary Information:**

The online version contains supplementary material available at 10.1007/s40520-024-02867-8.

## Introduction

Chronic lung diseases (CLDs), including chronic obstructive pulmonary disease (COPD), chronic bronchitis, and interstitial lung disease, are a major global health concern [1–3]. COPD alone affects approximately 300 million individuals and accounts for an estimated 3.2 million deaths annually, making it the third-leading cause of death worldwide [4, 5]. CLDs not only diminish quality of life but also impose a substantial healthcare burden on society [6]. As many CLDs are age related, the rapid aging of the global population will likely exacerbate the challenges posed by these diseases [7–9]. Identifying effective indicators of CLDs can aid in prevention and intervention, thereby reducing their societal burden.

Frailty, characterized by a reduction in an individual’s homeostatic reserve and increased vulnerability to acute stress, is associated with a higher risk of adverse health outcomes, including declines in endurance and cognitive function [10, 11]. The frailty index (FI) is a comprehensive tool that quantifies an individual’s frailty status based on indicators such as weight, fatigue, and nutritional status, with higher scores indicating more severe frailty [12]. Previous studies have shown that FI is an important factor in the progression or worsening of various diseases. For example, a high FI score is a risk factor for increased mortality in elderly patients with heart failure [13], while improvements in frailty reduce the risk of progression and death in cardiometabolic diseases [14]. FI is also a significant predictor of survival in patients undergoing surgery for bladder and ovarian cancers [15, 16], and frailty is linked to higher all-cause mortality in patients with lung cancer [17]. In hospitalized patients with acute exacerbation of interstitial lung disease, frailty is associated with more severe complications [18]. These findings suggest that FI plays an important role in lung-related diseases as well. However, no studies have explored the association between FI and the risk of various CLDs, requiring further research.

This study aims to address this gap by exploring the association between baseline frailty status and CLD risk using data from two large-scale national cohorts, the China Health and Retirement Longitudinal Study (CHARLS) and the English Longitudinal Study of Ageing (ELSA), which provide extensive follow-up data spanning several years to decades.

## Materials and methods

### Study population and ethical approval

This study included two prospective cohorts: the CHARLS and the ELSA [19, 20], both of which are nationally representative. Baseline data were collected from wave 1 of CHARLS (2011) and wave 2 of ELSA (2004–2005). Follow-up surveys continued through subsequent waves until the most recent data: wave 5 of CHARLS (2020) and wave 10 of ELSA (2020–2021). The inclusion criteria for study participants were as follows: (1) age 45 years or older; (2) complete baseline information; (3) sufficient data available to calculate the FI; (4) clear follow-up information, including follow-up status (CLD and survival) and follow-up year. The exclusion criteria were as follows: (1) presence of CLD at baseline; (2) insufficient information to calculate FI; (3) unclear follow-up information. Ethical approval was obtained from the Institutional Review Boards of Peking University (No. IRB00001052-11015) and the London Multi-Centre Research Ethics Committee (No. MREC/04/2/006) for the CHARLS and ELSA, respectively. Written informed consent was obtained from all participants [19, 20].

### Calculation of frailty index and establishment of frailty status

Frailty status in this study was determined using the FI, calculated from baseline data. Specifically, the FI was constructed according to standard procedures based on the accumulation of multiple health deficits. Following previous research [21, 22] and considering data availability, 33 indicators covering demographics, disease information, physical function, cognition, and other variables were selected (Supplementary Material 1). Each indicator was scored between 0 and 1, with 1 indicating the highest level of frailty. The final FI score for each participant was the arithmetic mean of these indicators. Participants were classified into frailty statuses based on FI, following a recognized method [21, 22]: robust (FI ≤ 0.10), frail (FI ≥ 0.25), and pre-frail (0.10 < FI < 0.25).

### Collection of covariate information

Covariates in this study included age, sex, smoking status, drinking status, educational level, and marital status. Smoking status was categorized as smokers (including former smokers) and non-smokers. Drinking status was divided into drinkers (those drinking more than once per month) and non-drinkers. Educational level was classified as below high school, high school, and college or above. Marital status included married and other statuses (single, separated, divorced, or widowed).

### Confirmation of CLD outcomes

The outcome variable was the incidence of CLD. CLD incidence was determined based on physician-diagnosed information provided by participants, including COPD, chronic bronchitis, emphysema, and pulmonary heart disease, excluding asthma and neoplastic diseases. The follow-up endpoint was the first occurrence of CLD, death, or the most recent follow-up date. Individuals without CLD who had died were treated as censored data (outcome variable as negative). Participants with missing CLD status information were excluded from this study.

### Calculation of propensity score weights for different FI groups

Propensity scores (PS) are used to reduce the impact of potential confounding factors when making between-group comparisons. By weighting or matching samples, PS ensures that covariates are similarly distributed across groups, improving comparability [23]. A generalized boosted model (GBM) makes it feasible to capture complex relationships between personal characteristics across multiple groups through an iterative process, identifying the optimal PS model that achieves the best balance between groups [24]. In this study, GBM was employed to estimate PS weights across the three FI groups (robust, pre-frail, and frail). This process was carried out using the “twang” package in R [24, 25]. The maximum number of iterations was set to 10,000, and the stopping point was determined by minimizing the absolute standardized mean difference (SMD) of effect sizes. The average treatment effect weight was used to estimate FI status effects for the overall population. Kaplan–Meier curve analysis, Cox regression, and accelerated failure time (AFT) analyses were performed using these weights. The balance of covariates between each pair of FI groups was evaluated using SMD before and after weighting. A balance between groups was considered achieved when SMD < 0.1.

### Sensitivity analysis

Sensitivity analyses were conducted to assess the robustness of the main findings. The AFT model is commonly employed in sensitivity analyses of the Cox model to confirm the robustness of the model results [26, 27], which was also applied in this study. The data distribution parameters for the AFT model (Weibull, log-normal, exponential, or logistic distribution) were determined based on the smallest Akaike Information Criterion value. Additionally, FI was treated as a continuous variable for the other sensitivity analysis using multivariate Cox regression rather than using fixed thresholds (i.e., 0.10 and 0.25) for FI status grouping.

### Other statistical analysis

Categorical variables were presented as counts and percentages. The Wilcoxon rank-sum test was used to assess differences in FI scores between groups with and without CLD. Kaplan–Meier curves and Cox regression analyses were conducted to examine the association between frailty status, covariates, and CLD risk. All statistical analyses were conducted using R software (version 4.2.2) and associated packages [25, 28–30], with statistical significance set at a two-sided *p*-value of < 0.05.

## Results

### Overview and baseline characteristics

After applying the inclusion and exclusion criteria, this study included a total of 9642 participants, with 8067 from the CHARLS cohort and 1575 from the ELSA cohort. Table 1 and Supplementary Material 2 present the baseline characteristics of both cohorts. In each, the frail group, compared to the robust and pre-frail groups, had more elder individuals (age not less than 65 years old), a larger proportion of females, more individuals with lower educational levels (below high school), and fewer married participants. The distribution of smoking and drinking statuses varied between the two cohorts. In the CHARLS cohort, the proportions of smokers and drinkers was lower across all frailty statuses, whereas the ELSA cohort showed the opposite trend (Table 1 and Supplementary Material 2).

**Table 1:** Baseline characteristics and standardized mean differences (SMDs) of the cohort from the China Health and Retirement Longitudinal Study

Characteristics	Robust (*N* = 1246)	Pre-frail (*N* = 5103)	Frail (*N* = 1718)	Maximum SMD	
				Unweighted	Weighted
Age (year)				0.777	0.010
<65	1095 (87.9%)	3822 (74.9%)	912 (53.1%)		
≥65	151 (12.1%)	1281 (25.1%)	806 (46.9%)		
Sex				0.533	0.005
Female	610 (49%)	3429 (67.2%)	1275 (74.2%)		
Male	636 (51%)	1674 (32.8%)	443 (25.8%)		
Smoking status				0.272	0.004
No	788 (63.2%)	3726 (73%)	1296 (75.4%)		
Yes	458 (36.8%)	1377 (27%)	422 (24.6%)		
Drinking status				0.099	0.014
No	1163 (93.3%)	4724 (92.6%)	1558 (90.7%)		
Yes	83 (6.7%)	379 (7.4%)	160 (9.3%)		
Education				0.477	0.011
Below high school	1027 (82.4%)	4653 (91.2%)	1652 (96.2%)		
High school	177 (14.2%)	397 (7.8%)	53 (3.1%)		
College or above	42 (3.4%)	53 (1%)	13 (0.8%)		
Marital status				0.410	0.001
Married	1141 (91.6%)	4471 (87.6%)	1329 (77.4%)		
Others	105 (8.4%)	632 (12.4%)	389 (22.6%)		

*Notes* The weights were derived using the “twang” package, which estimates propensity scores. For each group, the propensity score was compared to a pooled sample of individuals from other groups. This process generated weights used to balance the covariates across the groups. The maximum SMDs are presented in this table

### Evaluation of balance between FI groups

Before applying PS weighting, multiple clinical characteristics in the CHARLS cohort were imbalanced across the different FI groups (SMD > 0.1, Table 1). A similar imbalance was observed in the ELSA cohort (SMD > 0.1, Supplementary Material 2). After applying PS weights based on GMB, SMDs changed significantly in both cohorts (Supplementary Materials 3 and 4). Following weighting, clinical characteristics in both cohorts were balanced across FI groups (SMD < 0.1; Table 1 and Supplementary Material 2), allowing for appropriate comparisons between the groups.

### Association between FI and CLD Risk

**Fig. 1:**
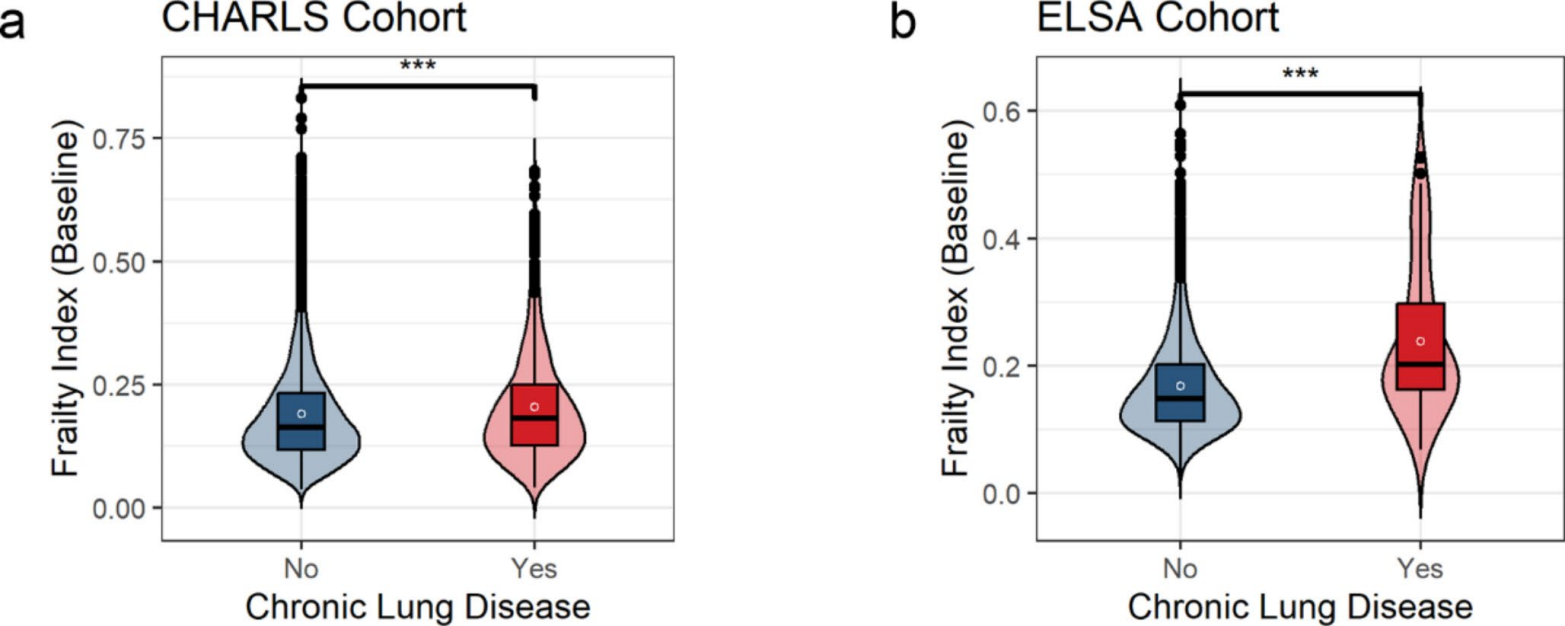
Differences in FI between groups with and without CLD. *P*-values were calculated based on Wilcoxon rank-sum tests. ^***^p < .001. CHARLS, China Health and Retirement Longitudinal Study. ELSA, English Longitudinal Study of Ageing

In the CHARLS cohort, participants with CLD exhibited higher FI scores than those without CLD (*p* < .05). This phenomenon was also observed in the ELSA cohort (*p* < .05, Fig. 1a and b), suggesting that FI may serve as a marker for CLD. To further analyze this relationship, Kaplan–Meier curves were used to examine the association between varying FI levels and CLD risk in both the CHARLS and ELSA cohorts.

**Fig. 2:**
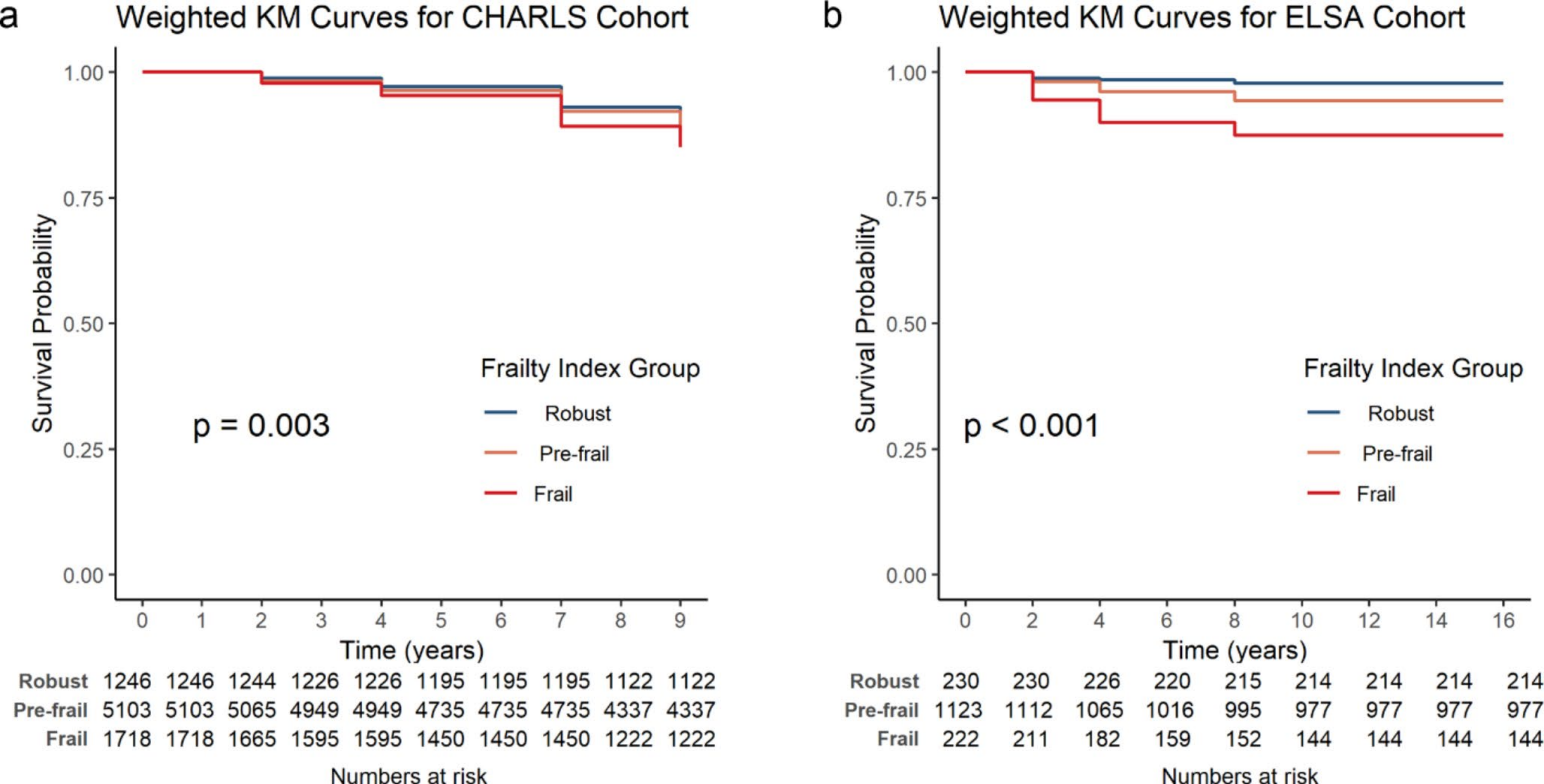
Weighted Kaplan–Meier curves analyzing the association between different FI levels and CLD risk in the CHARLS (**a**) and ELSA (**b**) cohorts

The CHARLS cohort revealed a significant association between FI and CLD risk (*p* < .05, Fig. 2a). Specifically, higher FI levels (frail and pre-frail vs. robust) were significantly associated with an increased risk of developing CLD, indicating that greater frailty corresponds to a heightened risk of CLD (*p* < .05, Fig. 2a). To validate these findings, the same analytical methods were applied to the ELSA cohort, which similarly indicated that higher FI levels were significantly associated with increased CLD risk (*p* < .05, Fig. 2b). Thus, results from both the CHARLS and ELSA cohorts suggest that FI is an important predictor of CLD risk.

### Association between FI, covariates, and CLD risk

To minimize potential confounding factors affecting the association between FI and CLD risk, multivariate Cox regression analyses were employed to assess the relationship between various characteristics and the hazard ratio (HR) of CLD. In the CHARLS cohort, 900 of 8067 participants (67,083 person-years) were diagnosed CLDs (Table 2). Age, sex, smoking status, drinking status, and education were not significantly associated with CLD risk (*p* > .05, Table 2). In contrast, marital status and frailty status emerged as strong independent predictors of CLD. Specifically, unmarried status (HR = 1.37, *p* = .039) was a significant risk factor for CLD compared to married status (Table 2). Additionally, frail individuals exhibited a higher risk of CLD (HR = 1.54, *p* = .003) compared to those classified as robust, while pre-frail status did not show a positive association (Table 2).

In the ELSA cohort, 95 of 1575 participants (22,344 person-years) were diagnosed with CLDs. Significant independent risk factors for CLD included age not less than 65 years old (HR = 2.24), smoking status (HR = 3.05), unmarried status (HR = 2.14), pre-frail status (HR = 2.60), and frail status (HR = 6.64) (*p* < .05; Supplementary Material 5). Thus, marital status and frail status were strong independent risk factors for CLD across both cohorts.

**Table 2:** Results of the multivariable Cox analysis examining the association between different frailty index (FI) levels and chronic lung disease (CLD) risk in China Health and Retirement Longitudinal Study

Characteristics	Number	CLD events	Person years	CLD incidence rate	Hazard ratio (95% CI)	*P* value
Age (year)						
<65	5829	660	49,743	13.27		
≥65	2238	240	17,340	13.84	1.01 (0.79–1.29)	0.942
Sex						
Female	5314	551	44,797	12.30		
Male	2753	349	22,286	15.66	1.10 (0.85–1.43)	0.477
Smoking status						
No	5810	583	48,964	11.91		
Yes	2257	317	18,119	17.50	1.29 (0.99–1.67)	0.056
Drinking status						
No	7445	811	62,192	13.04		
Yes	622	89	4891	18.20	1.25 (0.92–1.70)	0.146
Education						
Below high school	7332	820	60,825	13.48		
High school	627	67	5348	12.53	0.92 (0.64–1.33)	0.653
College or above	108	13	910	14.29	0.90 (0.45–1.79)	0.765
Marital status						
Married	6941	759	58,327	13.01		
Others	1126	141	8756	16.10	1.37 (1.02–1.85)	0.039^*^
FI group						
Robust	1246	119	10,771	11.05		
Pre-frail	5103	555	42,945	12.92	1.15 (0.89–1.49)	0.292
Frail	1718	226	13,367	16.91	1.54 (1.16–2.06)	0.003^*^

*Notes* CLD incidence rate was calculated per 1000 person-years. Hazard ratios and *p*-values were calculated using multivariable Cox regression analysis. ^*^P < .05

Stratification analysis was performed to further investigate the association between different FI statuses and CLD risk. In the CHARLS cohort, younger (< 65 years) frail individuals had a significantly higher risk of CLD (HR = 1.87), with similar findings for frail females (HR = 1.60), non-smokers (HR = 1.55), individuals with low education (below high school) (HR = 1.60), and married individuals (HR = 1.64) (*p* < .05; Table 3). Notably, pre-frail status was identified as a risk factor for CLD only among those younger than 65 years of age (*p* < .05; Table 3). In the ELSA cohort, frailty was strongly associated with CLD risk across different age groups (HR > 1.00, *p* < .05; Table 3). Frail individuals with smoking, drinking, different education levels (except for high school), and different marital statuses also exhibited a markedly increased risk of developing CLDs (HR > 1.00, *p* < .05; Table 3). Overall, frailty consistently emerged as a key predictor of CLD risk.

**Table 3:** Results of the stratification analysis examining the association between different FI status and CLD risk

Characteristics	HR (95% CI) for CHARLS cohort			HR (95% CI) for ELSA cohort	
	Pre-frail vs. robust	Frail vs. robust		Pre-frail vs. robust	Frail vs. robust
Age (year)					
<65	1.36 (1.07–1.73)^*^	1.87 (1.41–2.48)^*^		2.33 (0.69–7.84)	7.06 (1.92–25.95)^*^
≥65	0.79 (0.44–1.41)	0.94 (0.51–1.71)		2.87 (0.69–11.99)	5.63 (1.26–25.15)^*^
Sex					
Female	1.17 (0.79–1.73)	1.60 (1.06–2.43)^*^		NA	NA
Male	1.12 (0.84–1.49)	1.43 (0.98–2.08)		1.43 (0.54–3.78)	3.06 (0.96–9.76)
Smoking status					
No	1.11 (0.78–1.59)	1.55 (1.06–2.29)^*^		NA	NA
Yes	1.23 (0.88–1.70)	1.49 (0.99–2.24)		1.95 (0.74–5.13)	4.98 (1.77–14.02)^*^
Drinking status					
No	1.17 (0.88–1.55)	1.58 (1.16–2.15)^*^		NA	NA
Yes	0.96 (0.49–1.85)	1.12 (0.53–2.35)		1.97 (0.75–5.16)	4.13 (1.41–12.07)^*^
Education					
Below high school	1.18 (0.89–1.57)	1.60 (1.17–2.17)^*^		2.19 (0.76–6.31)	3.43 (1.11–10.58)^*^
High school	0.98 (0.52–1.86)	1.18 (0.46–3.05)		NA	NA
College or above	0.44 (0.13–1.52)	0.20 (0.02–1.94)		3.3 (0.41–26.25)	21.42 (2.56–179.30)^*^
Marital status					
Married	1.23 (0.95–1.59)	1.64 (1.22–2.19)^*^		3.35 (0.98–11.47)	6.51 (1.74–24.33)^*^
Others	0.83 (0.38–1.83)	1.11 (0.49–2.50)		1.58 (0.34–7.23)	5.41 (1.11–26.44)^*^

*Notes* Cox regression analysis was applied in this table. ^*^P < .05. NA denotes “not applicable” due to sparse data; for instance, in the ELSA cohort, there were no events in the female subgroup

### Sensitivity analysis

The sensitivity analysis further confirmed the main Cox regression results. Using the AFT method, both the CHARLS and ELSA cohorts demonstrated that pre-frail and frail statuses are significant risk factors for CLD (time ratio < 1.00, *p* < .05; Supplementary Material 6). All significant results from the Cox analysis (Table 2 and Supplementary Material 5) were verified using the AFT method (Supplementary Material 6). Furthermore, where FI was treated as a continuous variable, it remained an independent prognostic factor for CLD risk (HR > 1.00, *p* < .05; Supplementary Material 7). Thus, the sensitivity analysis did not substantially alter the significant associations observed in the primary analysis.

## Discussion

This study included two national prospective cohorts, comprising a total of 9642 samples (8067 from the CHARLS cohort and 1575 from the ELSA cohort), to evaluate the association between FI and the risk of various CLDs. Consistent findings across both cohorts indicated that frailty and unmarried status are independent risk factors for CLD. These results may aid in the timely identification, monitoring, and intervention of potential patients with CLD.

A high baseline FI score was associated with an increased risk of developing CLD during follow-up. As the global aging trend intensifies, frailty is becoming increasingly common and is a growing concern for researchers. A high FI is not only a prognostic indicator for many malignant diseases, such as lung cancer and colorectal cancer [31–33], but also a significant risk factor for various non-malignant diseases, including heart disease [13, 14]. In CLD-related research, Nishimura et al. [34] reported that frail individuals within the COPD patient population have a higher risk of acute exacerbations and hospitalization than their robust counterparts. Van and colleagues [18] found that a high FI is associated with major complications in hospitalized patients with acute exacerbations of interstitial lung disease. These studies highlight the prognostic value of FI in CLD.

To the best of our knowledge, no previous studies have explored the use of FI in assessing CLD risk in the general population. Our study addresses this gap for the first time, revealing that individuals with CLD have higher FI scores than those without CLD. More importantly, individuals classified as frail at baseline are more likely to develop CLD compared to robust individuals, a finding that was consistent across most stratified variables. These results are supported by two large prospective cohorts from China and the United Kingdom and validated through sensitivity analysis, demonstrating the robustness of our findings. Furthermore, based on the ELSA cohort, pre-frailty is also a risk factor for CLD compared to robust individuals, although the HR for pre-frailty is lower than that for frailty, suggesting an increased risk of developing CLD during the transition from robust to pre-frail to frail states. Additionally, we found that unmarried status is associated with an increased risk of CLD, possibly influenced by physiological and psychological factors [35–37]. In summary, our findings suggest that FI and unmarried status are independent risk factors for CLD.

Risk factors for CLD may vary among different populations. In our study, individuals in the ELSA cohort with higher age or smoking status exhibited an increased risk of CLD. This trend is understandable, as advanced age, especially among the elderly, typically indicates poorer physiological function, making individuals more susceptible to CLD [38, 39]. Smoking is known to contribute to CLDs, such as interstitial lung disease and COPD [40, 41], through various mechanisms, including the disruption of alveolar macrophages, damage to the airway epithelial barrier, and adverse effects on mitochondrial function in cells [40, 42, 43]. However, in the CHARLS cohort, no associations were observed between smoking status or age and CLD risk. This discrepancy may be due to differences in social factors and physiological characteristics between Asian (CHARLS cohort) and European populations (ELSA cohort), warranting further research.

This study has several limitations. First, the determination of CLD was based on self-reported physician diagnoses rather than medical records, which may introduce bias. However, the large sample size and dual-cohort design help mitigate this potential bias. Second, the analysis only included individuals with complete baseline information and sufficient data to calculate the FI, potentially leading to selection bias. Third, although we adjusted for various confounding factors, some unconsidered confounders, such as physiological differences and dietary habits between groups, may require further investigation. Fourth, due to sparse data, not all subgroup analyses were performed for every stratification in the ELSA cohort, which may have contributed to the loss of some significant findings.

In conclusion, this study identifies frailty status as an important indicator for CLD risk, with a high FI associated with increased CLD risk. Focusing on frailty status may facilitate the development of prevention strategies for CLD and enable targeted interventions.

## Electronic Supplementary Material

Below is the link to the electronic supplementary material.

Supplementary Material 1

Supplementary Material 2

Supplementary Material 3

Supplementary Material 4

Supplementary Material 5

Supplementary Material 6

Supplementary Material 7

## Data Availability

The data that support the findings of this study are available in China Health and Retirement Longitudinal Study (https://charls.pku.edu.cn/index.htm) and English Longitudinal Study of Ageing (https://www.elsa-project.ac.uk/).
