# Supplementary material for: Association between frailty status and risk of chronic lung disease: an analysis based on two national prospective cohorts
Source: Aging Clin Exp Res. 2024 Nov 9;36(1):215. doi: 10.1007/s40520-024-02867-8 (PMC11550224; doi:10.1007/s40520-024-02867-8)
Supplement: Supplementary file 1 — Supplementary Material 1 [file 40520_2024_2867_MOESM1_ESM.pdf]

Supplementary Material 1. The 33 items used to construct the frailty index scores.

Items			Cut-off value
**No**	**CHARLS**	**ELSA**	
1	Self-reported vision problems		CHARLS: 1 = yes, 2 = no; ELSA: 5 = yes, 4- = no
2	Self-reported hearing problems		CHARLS: 1 = yes, 2 = no; ELSA: 5 = yes, 4- = no
3	Ever diagnosed high blood pressure		CHARLS: 1 = yes, 2 = no; ELSA: 1, 2 = yes, 0 = no
4	Ever diagnosed diabetes		CHARLS: 1 = yes, 2 = no; ELSA: 1, 2 = yes, 0 = no
5	Ever diagnosed cancer		CHARLS: 1 = yes, 2 = no; ELSA: 1, 2 = yes, 0 = no
6	Ever diagnosed heart disease		CHARLS: 1 = yes, 2 = no; ELSA: 1, 2 = yes, 0 = no
7	Ever diagnosed stroke		CHARLS: 1 = yes, 2 = no; ELSA: 1, 2 = yes, 0 = no
8	Ever diagnosed emotional, nervous, or psychiatric problems		CHARLS: 1 = yes, 2 = no; ELSA: 1, 2 = yes, 0 = no
9	Ever diagnosed memory-related disease		CHARLS: 1 = yes, 2 = no; ELSA: 1, 2 = yes, 0 = no
10	Ever diagnosed arthritis or rheumatism		CHARLS: 1 = yes, 2 = no; ELSA: 1, 2 = yes, 0 = no
11	Mobility: difficulty getting up from chair after sitting long periods		CHARLS: 2+ = yes, 1 = no; ELSA: 1 = yes, 0 = no
12	Mobility: difficulty climbing several flights stairs without resting		CHARLS: 2+ = yes, 1 = no; ELSA: 1 = yes, 0 = no
13	Mobility: difficulty stooping, kneeling or crouching		CHARLS: 2+ = yes, 1 = no; ELSA: 1 = yes, 0 = no
14	Mobility: difficulty reaching or extending arms above shoulder level		CHARLS: 2+ = yes, 1 = no; ELSA: 1 = yes, 0 = no
15	Have difficulty lifting or carrying weights over 10 jin / pounds		CHARLS: 2+ = yes, 1 = no; ELSA: 1 = yes, 0 = no
16	Have difficulty picking up a small / 5p coin from a table		CHARLS: 2+ = yes, 1 = no; ELSA: 1 = yes, 0 = no
17	Difficulty dressing		CHARLS: 2+ = yes, 1 = no; ELSA: 1 = yes, 0 = no
18	Difficulty bathing or showering		CHARLS: 2+ = yes, 1 = no; ELSA: 1 = yes, 0 = no
19	Difficulty eating		CHARLS: 2+ = yes, 1 = no; ELSA: 1 = yes, 0 = no
20	Difficulty using the toilet, including getting up or down		CHARLS: 2+ = yes, 1 = no; ELSA: 1 = yes, 0 = no
21	Difficulty preparing a hot meal		CHARLS: 2+ = yes, 1 = no; ELSA: 1 = yes, 0 = no
22	Difficulty shopping for groceries		CHARLS: 2+ = yes, 1 = no; ELSA: 1 = yes, 0 = no
23	Difficulty managing money		CHARLS: 2+ = yes, 1 = no; ELSA: 1 = yes, 0 = no
24	Difficulty taking medications		CHARLS: 2+ = yes, 1 = no; ELSA: 1 = yes, 0 = no
25	Difficulty getting in and out of bed		CHARLS: 2+ = yes, 1 = no; ELSA: 1 = yes, 0 = no
26	Body mass index		CHARLS: weight/(height^2); ELSA: BMI
27	Mean systolic blood pressure		Measurement mean
28	Mean diastolic blood pressure		Measurement mean
29	Mean pulse		Measurement mean
30	Mean peak expiratory flow reading		Measurement mean

31	Mean grip strength	Measurement mean
32	Cognition: (memory test score + orientation test score) / 14	Continuous, ranging from 0 to 1
33	Depression: CESD-10 or Depression: CESD-8	Continuous, ranging from 0 to 1
