# Supplementary material for: Association between frailty status and risk of chronic lung disease: an analysis based on two national prospective cohorts
Source: Aging Clin Exp Res. 2024 Nov 9;36(1):215. doi: 10.1007/s40520-024-02867-8 (PMC11550224; doi:10.1007/s40520-024-02867-8)
Supplement: Supplementary file 2 — Supplementary Material 2 [file 40520_2024_2867_MOESM2_ESM.pdf]

Supplementary Material 2. Baseline characteristics and standardized mean differences (SMDs) of the cohort from the English Longitudinal Study of Ageing Study.

Characteristics	Robust (N=230)	Pre-frail (N=1123)	Frail (N=222)	Maximum SMD	
				Unweighted	Weighted
Age (year)				0.504	0.017
<65	175 (76.1%)	788 (70.2%)	117 (52.7%)		
⩾65	55 (23.9%)	335 (29.8%)	105 (47.3%)		
Sex				0.618	0.008
Female	74 (32.2%)	603 (53.7%)	140 (63.1%)		
Male	156 (67.8%)	520 (46.3%)	82 (36.9%)		
Smoking status				0.260	0.022
No	103 (44.8%)	475 (42.3%)	71 (32%)		
Yes	127 (55.2%)	648 (57.7%)	151 (68%)		
Drinking status				0.536	0.041
No	26 (11.3%)	190 (16.9%)	71 (32%)		
Yes	204 (88.7%)	933 (83.1%)	151 (68%)		
Education				0.461	0.016
Below high school	90 (39.1%)	579 (51.6%)	138 (62.2%)		
High school	22 (9.6%)	114 (10.2%)	19 (8.6%)		
College or above	118 (51.3%)	430 (38.3%)	65 (29.3%)		
Marital status				0.282	0.052
Married	189 (82.2%)	863 (76.8%)	156 (70.3%)		
Others	41 (17.8%)	260 (23.2%)	66 (29.7%)		

Notes: The weights were derived using the "twang" package, which estimates propensity scores. For each group, the propensity score was compared to a pooled sample of individuals from other groups. This process generated weights used to balance the covariates across the groups. The maximum SMDs are presented in this table.
