# Supplementary material for: Association between frailty status and risk of chronic lung disease: an analysis based on two national prospective cohorts
Source: Aging Clin Exp Res. 2024 Nov 9;36(1):215. doi: 10.1007/s40520-024-02867-8 (PMC11550224; doi:10.1007/s40520-024-02867-8)
Supplement: Supplementary file 3 — Supplementary Material 3 [file 40520_2024_2867_MOESM3_ESM.pdf]

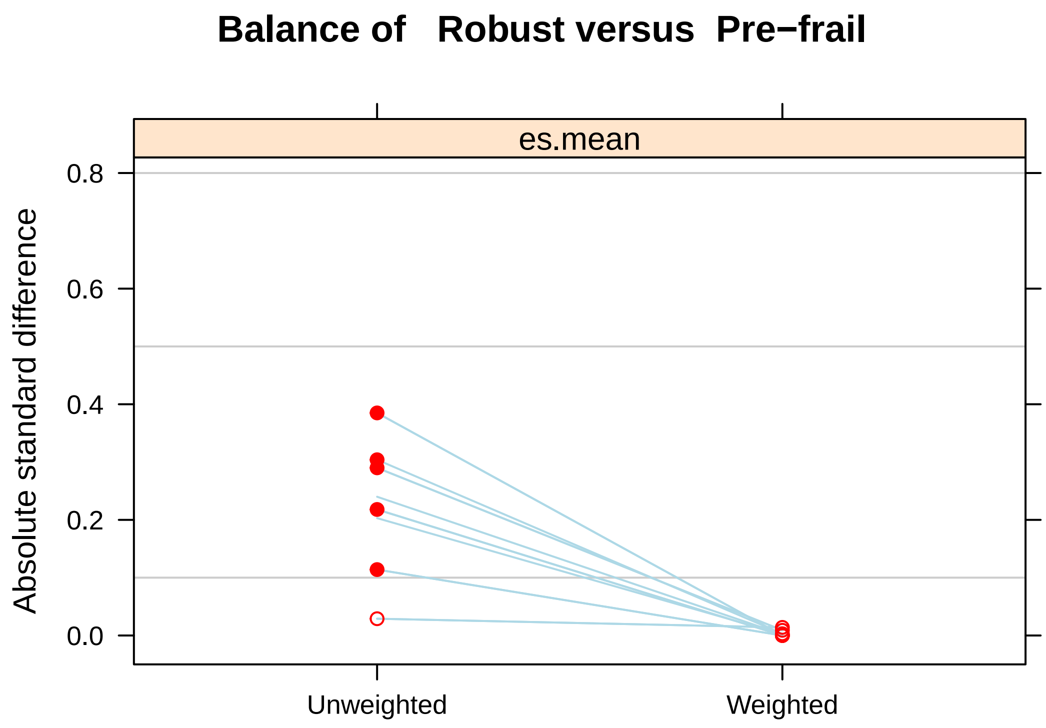

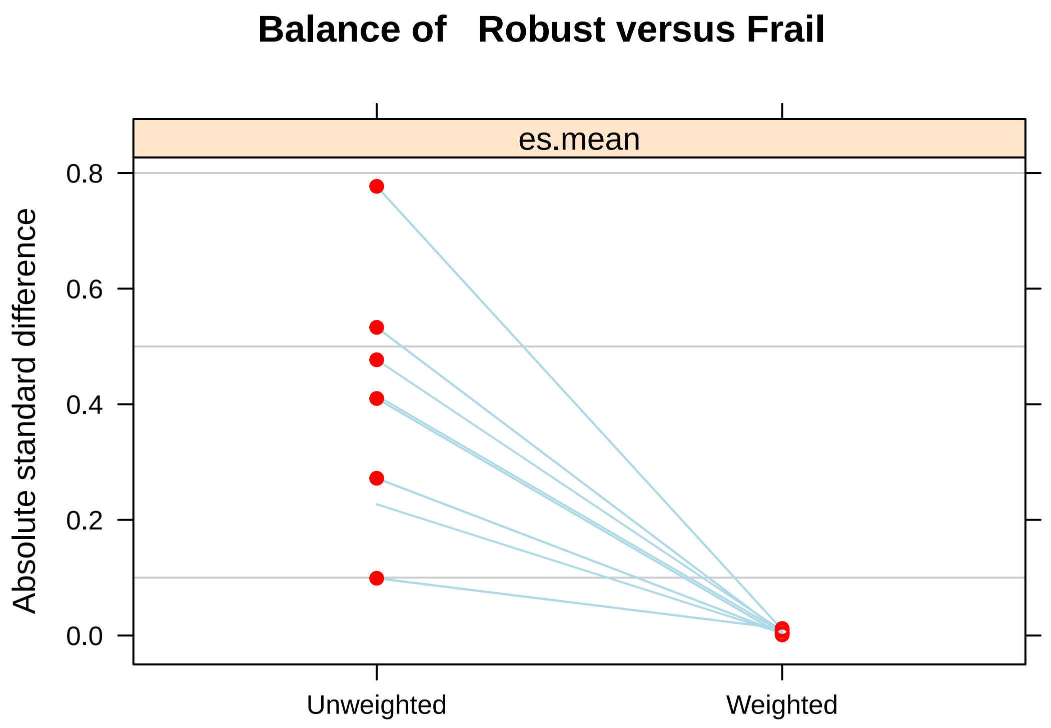

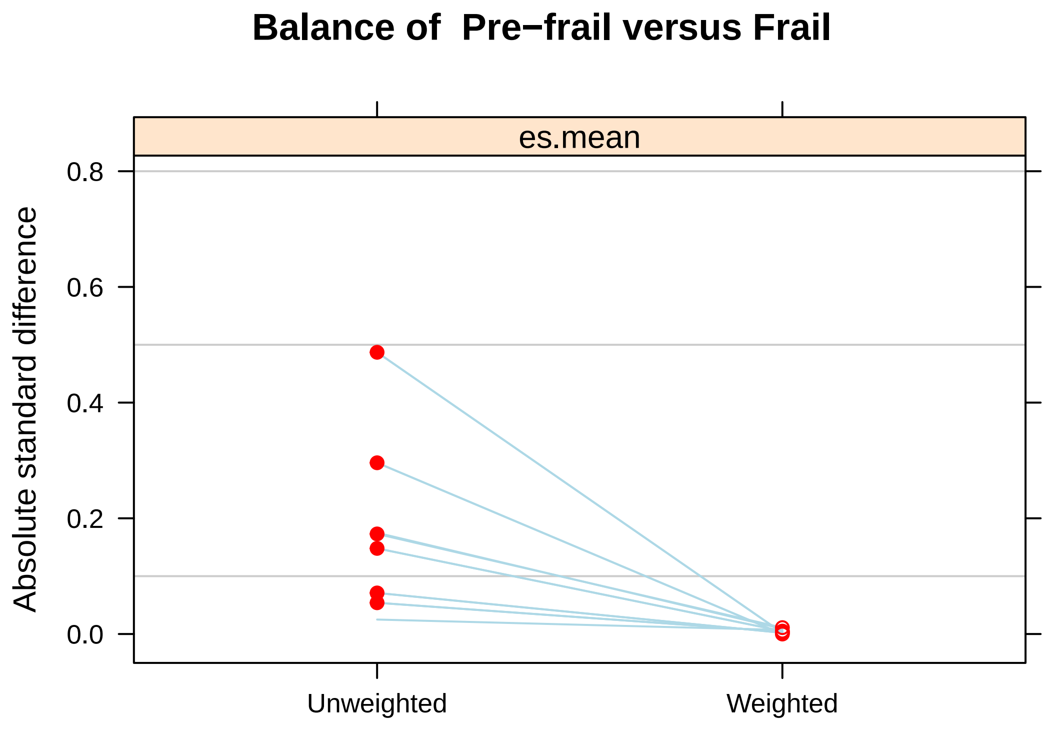

Supplementary Material 3. Graphical assessments of the balance of the CHARLS cohort. The panels compare the absolute standardized mean differences (SMDs) between groups on pretreatment covariates, both before and after weighting. After weighting, the maximum SMD decreases across all pretreatment covariates. Statistically significant differences (prior to taking the maximum across groups) from t-tests or chi-squared statistics, based on the pairwise minimum p-values, are indicated by solid circles.
