# Supplementary material for: Association between frailty status and risk of chronic lung disease: an analysis based on two national prospective cohorts
Source: Aging Clin Exp Res. 2024 Nov 9;36(1):215. doi: 10.1007/s40520-024-02867-8 (PMC11550224; doi:10.1007/s40520-024-02867-8)
Supplement: Supplementary file 5 — Supplementary Material 5 [file 40520_2024_2867_MOESM5_ESM.pdf]

Supplementary Material 5. Results of the multivariable Cox analysis examining the association between different FI levels and CLD risk in the English Longitudinal Study of Ageing Study.

Characteristics	Number	CLD events	Person years	CLD incidence rate	Hazard ratio (95% CI)	P value
Age (year)						
<65	1080	42	16386	2.56		
≥65	495	53	5958	8.90	2.24 (1.24-4.07)	0.008*
Sex						
Female	817	46	11923	3.86		
Male	758	49	10421	4.70	1.68 (0.97-2.91)	0.063
Smoking status						
No	649	21	9566	2.20		
Yes	926	74	12778	5.79	3.05 (1.60-5.82)	0.001*
Drinking status						
No	287	27	3751	7.20		
Yes	1288	68	18593	3.66	0.65 (0.35-1.19)	0.164
Education						
Below high school	807	65	11039	5.89		
High school	155	11	2198	5.00	0.83 (0.32-2.18)	0.707
College or above	613	19	9107	2.09	0.84 (0.42-1.68)	0.613
Marital status						
Married	1208	61	17390	3.51		
Others	367	34	4954	6.86	2.14 (1.16-3.98)	0.015*
FI group						
Robust	230	5	3498	1.43		
Pre-frail	1123	59	16255	3.63	2.60 (1.00-6.78)	0.050*
Frail	222	31	2591	11.96	6.64 (2.43-18.17)	<0.001*

Notes: CLD incidence rate was calculated per 1000 person years. Hazard ratios and *p*-values were calculated using multivariable Cox regression analysis. $^{*}P < 0.05$.
