# Supplementary material for: Association between frailty status and risk of chronic lung disease: an analysis based on two national prospective cohorts
Source: Aging Clin Exp Res. 2024 Nov 9;36(1):215. doi: 10.1007/s40520-024-02867-8 (PMC11550224; doi:10.1007/s40520-024-02867-8)
Supplement: Supplementary file 6 — Supplementary Material 6 [file 40520_2024_2867_MOESM6_ESM.pdf]

Supplementary Material 6. Sensitivity analysis (accelerated failure time model) of the association between different FI levels and CLD risk in two cohorts.

Characteristics	CHARLS cohort		ELSA cohort	
	**Time ratio (95% CI)**	**P value**	**Time ratio (95% CI)**	**P value**
Age group (≥ 65 years vs. <65 years)	0.99 (0.95-1.02)	0.462	0.34 (0.24-0.46)	<0.001*
Sex (male vs. female)	0.95 (0.91-1.00)	0.051	0.46 (0.34-0.64)	<0.001*
Smoking status (yes vs. no)	0.90 (0.85-0.94)	<0.001*	0.24 (0.16-0.34)	<0.001*
Drinking status (yes vs. no)	0.91 (0.86-0.96)	0.001*	1.68 (1.15-2.43)	0.007
Education (college or above vs. below high school)	1.05 (0.90-1.22)	0.530	1.54 (1.10-2.15)	0.012*
Education (high school vs. below high school)	1.04 (0.98-1.11)	0.199	1.34 (0.77-2.35)	0.304
Marital status (other vs. married)	0.87 (0.83-0.91)	<0.001*	0.37 (0.27-0.52)	<0.001*
FI group (pre-frail vs. robust)	0.94 (0.90-0.98)	0.003*	0.30 (0.19-0.48)	<0.001*
FI group (frail vs. robust)	0.82 (0.79-0.86)	<0.001*	0.09 (0.05-0.14)	<0.001*

Notes: $^{*}P < 0.05$.
