# Supplementary material for: Association between frailty status and risk of chronic lung disease: an analysis based on two national prospective cohorts
Source: Aging Clin Exp Res. 2024 Nov 9;36(1):215. doi: 10.1007/s40520-024-02867-8 (PMC11550224; doi:10.1007/s40520-024-02867-8)
Supplement: Supplementary file 7 — Supplementary Material 7 [file 40520_2024_2867_MOESM7_ESM.pdf]

Supplementary Material 7. Sensitivity analysis (multivariable Cox analysis) examining the association between different FI levels and CLD risk in two cohorts.

Characteristics	CHARLS cohort		ELSA cohort	
	**HR (95% CI)**	**P value**	**HR (95% CI)**	**P value**
Age group (≥ 65 years vs. <65 years)	0.99 (0.77-1.27)	0.910	2.58 (1.41-4.72)	0.002*
Sex (male vs. female)	1.10 (0.85-1.42)	0.483	1.68 (0.96-2.96)	0.070
Smoking status (yes vs. no)	1.30 (1.01-1.68)	0.043*	2.58 (1.35-4.96)	0.004*
Drinking status (yes vs. no)	1.24 (0.92-1.68)	0.162	0.83 (0.46-1.51)	0.544
Education (high school vs. below high school)	0.93 (0.64-1.35)	0.704	0.87 (0.32-2.32)	0.777
Education (college or above vs. below high school)	0.95 (0.49-1.86)	0.883	0.80 (0.40-1.62)	0.541
Marital status (other vs. married)	1.36 (1.01-1.84)	0.043*	2.10 (1.10-4.01)	0.025*
Frailty index (per one standard deviation)	1.19 (1.10-1.29)	<0.001*	1.68 (1.44-1.97)	<0.001*

Notes: Frailty index is used as the continuous variable.
